# Proportion of extended-spectrum ß-lactamase-producing *Enterobacteriaceae *in community setting in Ngaoundere, Cameroon

**DOI:** 10.1186/1471-2334-12-53

**Published:** 2012-03-09

**Authors:** Carine Magoué Lonchel, Cécile Meex, Joseph Gangoué-Piéboji, Raphaël Boreux, Marie-Claire Okomo Assoumou, Pierrette Melin, Patrick De Mol

**Affiliations:** 1 Laboratory of Medical Microbiology, University of Liège, CHU Sart-Tilman (B23), B-4000 Liege, Belgium; 2 Institute of Medical Research and Medicinal Plant Studies, CRPMT, P.O. Box 8404, Yaounde, Cameroon; 3 Department of virology, Faculty of Medicine and Biomedical Sciences, University of Yaounde I, P.O. Box 8445, Yaounde, Cameroon

## Abstract

**Background:**

There is no information regarding the resistance mechanisms of extended-spectrum ß-lactamase (ESBL)-producing *Enterobacteriaceae *in community setting in Cameroon. The current study aimed to determine the proportion of ESBLs in *Enterobacteriaceae *isolated in the community and to analyse some risk factors associated with ESBL carriage.

**Methods:**

Faecal samples were collected from 208 different outpatients and 150 healthy student volunteers between 3 January and 3 April 2009. Enterobacterial isolates resistant to third-generation cephalosporins were screened for ESBL production by the double-disk synergy test. Presumptive ESBL-producing isolates with positive synergy test were identified by Mass Spectrometry using the BioTyper MALDI-TOF. For such ESBL positive isolates, antibiotic susceptibility was determined by the Vitek 2 system. PCR and sequencing were performed for the detection of different types of ESBL genes in presumptive ESBL-producing isolates. Statistical methods were used for the univariate calculation of risk factors.

**Results:**

During the study period, a total of 358 faecal samples were analysed; 58 of such samples (16%) showed an ESBL phenotype and were confirmed by PCR. The proportion of ESBL producers in faecal carriage was statistically different between outpatients and student volunteers (23.1% vs. 6.7%: *p *< 0.000). According to a univariate analysis, previous use of antibiotics (ciprofloxacin) appeared to be a risk factor for ESBL carriage (*p *< 0.05).

*Escherichia coli *was the species most frequently isolated among the ESBL producers in outpatients (66.7%) and student volunteers (90%). Isolates showed additional resistance to gentamicin, ciprofloxacin and trimethoprim/sulfamethoxazole but none of them was resistant to temocillin, amikacin or meropenem. Most of the strains (97%) produced a CTX-M group 1 enzymes [CTX-M-15 (98%) or CTX-M-1 (2%)] and the remaining strains produced SHV-12 enzyme (3%).

**Conclusions:**

The use of drugs such as amoxicillin, ciprofloxacin and trimethoprim/sulfamethoxazole does not seem appropriate for empirical treatment because of emerging resistance. The implementation in Cameroon or in other African countries of methods of screening ESBL-producing organisms in routine laboratories is of great importance in order for us to offer patients appropriate treatment and for infection control efforts to succeed.

## Background

Since the early 1980s, third-generation cephalosporins have become an important tool in the treatment of severe bacterial infections. Unfortunately, extended-spectrum ß-lactamases (ESBLs), responsible for resistance against almost all penicillins, cephalosporins (except cephamycins), and other monobactams, have been acquired by a great number of bacterial species [1]. ESBL-producing *Enterobacteriaceae *have been responsible for many local, national and international outbreaks and have become a serious problem in hospitalized patients [2]. Recent studies suggest that ESBL-producing *Enterobacteriaceae *should not be considered as exclusively nosocomial pathogens. ESBL-producing *Enterobacteriaceae *have been reported to cause urinary tract infections and bacteraemia in outpatients [2,3]. Since 2001, reports of community-acquired infections caused by ESBL-producing *Enterobacteriaceae *have emerged worldwide [3-12]. However, in African countries, data remain scarce; only a few studies have been conducted in community or hospital settings during nonoutbreak situations [4,13-16].

A study in Egypt found a proportion of 11% of ESBL-producing *Enterobacteriaceae *in a community setting [17], while a study in South Africa found a proportion of 7% [18]. In Cameroon, previous reports of SHV-12 and CTX-M-15 have been described [13,16]. The proportion of ESBLs in *Enterobacteriaceae *isolated from inpatients at Yaounde Central Hospital in Cameroon between 1995 and 1998 was found to be 12% [13]. However, no study on extended-spectrum ß-lactamase (ESBL)-producing *Enterobacteriaceae *has been performed in the community. As the first survey of its kind in Cameroon, the current study was conducted to determine the proportion of ESBLs in *Enterobacteriaceae *isolated in the community and to analyse some risk factors associated with ESBL carriage.

## Methods

### Study design

This study was conducted in the town of Ngaoundere, the capital of the Adamawa Province in Cameroon. In this part of the country, the main language is "Fulfulde" and the French language dominates as the language of instruction in schools. We were assisted in our interviews with patients by laboratory technicians who were suitably trained to conduct interviews. During a period of 3 months (between 3 January and 3 April 2009), a total of 358 faecal samples were collected from two population groups: 208 outpatients (who visited the bacteriology laboratory of Ngaoundere Protestant Hospital with a stool sample requested by their general practitioner) and 150 healthy student volunteers (the student volunteers at the University of Ngaoundere were attending the clinic for their annual medical examination. The volunteer subjects were provided with containers to collect their stool and were told to bring a fresh sample, preferably collected and delivered to the laboratory within a day). Of the outpatients, 6% had come for a planned medical visit and 13% were suffering from acute gastroenteritis. However, for 81% of the outpatients, there was insufficient information included in the patient record to enable us to define the exact reason for the consultation.

All samples were collected and refrigerated until processing. A questionnaire was designed and used to obtain demographic information and clinical data on the patient (age, gender, antibiotic treatment during the past 3 months and any hospital stays in the previous year).

### Detection of ESBL-producing isolates in faecal samples

A total of 0.5 g of each faecal sample was suspended in 5 mL of sterile saline, and aliquots of 50 μl were streaked onto two selective media, Drigalski and MacConkey agars, supplemented respectively with cefotaxime (1.5 mg/L) and ceftazidime (2 mg/L), enabling the detection of Gram-negative bacteria resistant to these antibiotics. Plates were incubated for 24 h at 35°C. One colony representing each distinct colonial morphotype was subcultured on MacConkey agar plates and was analysed further. All isolates that showed growth were screened for ESBL production by using both the resistance phenotype and the combined double-disk synergy test with conventional amoxicillin/clavulanate (10 μg of clavulanic acid), 30 μg cefotaxime, 30 μg ceftazidime and 30 μg cefepime disks (BBL, USA), which were applied at a 30 mm distance from amoxicillin/clavulanate [19].

### Identification and antimicrobial susceptibility testing

Only the isolates with a positive synergy test - the "presumptive ESBL producers" - were identified by Mass Spectrometry using the BioTyper MALDI-TOF (Bruker). For such ESBL positive isolates, antibiotic susceptibility to the following drugs was determined with the Vitek 2 system (bioMérieux, France): temocillin, meropenem, amikacin, gentamicin, ciprofloxacin, nitrofurantoin and trimethoprim/sulfamethoxazole. The results enabled us to define the isolates as being susceptible or resistant according to the criteria recommended by the Clinical and Laboratory Standards Institute [20]. All the presumptive ESBL producers were further analysed by PCR aimed at detecting ESBL genes (one isolate of each morphotype from each patient).

### Automated DNA isolation

Bacteria were grown overnight on a MacConkey agar plates at 35°C. Subsequently, colonies were picked up using a sterile 1 μl plastic loop and transferred into 400 μl sterile water. Total DNA was extracted with the Maxwell automat (Promega, USA) according to the manufacturer's protocol. DNA was eluted in a final volume of 200 μl. The extracted DNA was stored at -70°C for further analysis.

### PCR detection and sequencing of ESBLs

All isolates were initially screened for the presence of *bla*_TEM_, *bla*_SHV _and *bla*_OXA_-like genes using a multiplex PCR protocol previously described by Dallenne *et al. *[21]. All isolates were further screened for the presence of *bla*_CTX-M _carrying genes belonging to the CTX-M groups (i.e. CTX-M group1, 2, 8, 9 and 25) using a simplex PCR according to a previously published method [22]. Specific PCR amplification and DNA sequencing of the PCR products were used to determine whether the *bla*_TEM_, *bla*_SHV_, *bla*_OXA _and *bla*_CTX-M _groups were present and to characterize the type of ß-lactamase belonging to each familiy. PCR products (multiplex and simplex PCR) were visualized after electrophoresis at 100 V for 1 h on a 2% agarose gel containing ethidium bromide. A 100 bp DNA ladder (Promega, USA) was used as a marker size. PCR products were purified using the Wizard^® ^kit (Promega, USA) and sequenced using a 3100 ABI Prism Genetic Analyser (Applied Biosystems). Sequence alignment and analyses were performed online using the BLAST program available at the National Center for Biotechnology Information web page http://www.ncbi.nlm.nih.gov.

### Statistical analysis

Data was analysed with Epi Info version 3.5.3 (Centers for Disease Control and Prevention, Atlanta, GA, USA). We used conditional logistic regression analysis for the univariate calculation of risk factors and odds ratios (OR) with 95% confidence intervals (CI). A value of *p *< 0.05 was considered to be statistically significant. MIC analyses were carried out using the WHONET 5 software (World Health Organization, Geneva, Switzerland).

### Ethical clearance

The study was approved by the ethics committee of the University of Liège. The protocol was reviewed and accepted by the authorities of the hospitals in Cameroon (this allowed us to conduct our study). Informed consent was obtained from all patients before enrolment.

## Results

### Characteristics of the population

The mean age of the 208 outpatients was 36.9 ± 5.1 years; of these, 88 (42%) were male. The mean age of the student volunteers was 24.7 ± 7.3 years and 99 (66%) of these were male.

Of the outpatients, 16 (8%) had been hospitalized in the previous year and 44/208 (21%) had received antibiotics within the 3 months prior to inclusion in the study: 16 (36%) had received amoxicillin, 19 (43%) ciprofloxacin and the remaining 9 (21%) had received other antibiotics. Among the student volunteers, a single student had been hospitalized in the previous year and 15 (10%) patients had received antibiotics within the 3 months before sampling: 7 (47%) had received amoxicillin, 2 (13%) ciprofloxacin and the remaining 6 (40%) had received other antibiotics (see Table 1).

**Table 1:** Characteristics of the 358 patients included in the study

Characteristics	Outpatients N = 208 (%)	Student volunteers N = 150 (%)
Age (Mean, SD)	36.9,15.12	24.7,7.3

Male gender	88 (42)	99 (66)

Hospitalization (in previous year)		

yes	16 (8)	1 (1)

no	91 (44)	149 (99)

unknown	101 (48)	0

Antibiotic use (< 3 months)		

yes	44 (21)	15 (10)

no	48 (23)	132 (88)

unknown	116 (56)	3 (2)

Antibiotics used		

amoxicillin	14	7

ciprofloxacin	19	2

Trimethroprim/sulfamethoxazole	5	/

Other drugs	6	6

### Proportion of ESBL producers

During the study period, a total of 358 stool samples from outpatients and student volunteers were analysed; 58 of such samples (16%) contained isolates (1 isolate per patient) which grew on both selective media and were "presumptive ESBL producers". Of the 58 isolates, 48 were recovered from outpatients and 10 from student volunteers. All presumptive ESBLs were confirmed as ESBL producers by PCR. The proportion of ESBL-producing *Enterobacteriaceae *in outpatients was 23.1% (48/208) and 6.7% (10/150) in student volunteers. The proportion of ESBL producers in faecal carriage was statistically different between outpatients and student volunteers (23.1% vs. 6.7%: *p *< 0.000). A univariate analysis comparing subjects' ESBL positive and negative isolates identified no specific factor as being associated with ESBL carriage in both populations (Table 2). However, the previous use of antibiotics was different for both populations (*p *< 0.000) and the previous use of ciprofloxacin appeared to be a risk factor for ESBL carriage (*p *< 0.05) (Table 3).

**Table 2:** Analysis of risk factors for ESBL carriage in univariate analysis

	Outpatients			Student volunteers		
**Covariate**	**ESBL-positive**	**ESBL-negative**	***P value***	**ESBL-positive**	**ESBL-negative**	***P value***
				
	**(n = 48)**	**(n = 160)**		**(n = 10)**	**(n = 140)**	

Age (Mean, SD)	37.6,15.5	36.7,15	0.744	27.2,8.9	24.5,7.1	0.284

Male gender	21	67	0.817	7	92	0.782

Antibiotic use (< 3 months)						

yes/no	11/8	33/40	0.212	1/9	14/123	0.982

Hospitalization (previous year)						

yes/no	4/17	12/74	0.544	0/10	1/139	/

**Table 3:** Univariate analysis of antibiotics used among outpatients and student volunteers

Risk factors	Outpatients	Student volunteers	*P value*
Previous use of antibiotics			

yes/no	44/48	15/132	0.000

Previous use of ciprofloxacin			

yes/no	19/25	2/13	0.037

### Bacterial identification and antimicrobial susceptibility

For outpatients, the most frequent species isolated among the ESBL producers was *Escherichia coli *(66.7%), followed by *Enterobacter cloacae *(18.8%), *Citrobacter freundii *(10.4%) and *Klebsiella pneumoniae *(4.2%). Among student volunteers, of the 10 ESBL-producing strains, 9 were *Escherichia coli *(90%) and 1 strain was *Klebsiella pneumoniae *(10%).

Antibiotic susceptibility was determined for 7 agents. All isolates were susceptible to temocillin, meropenem and amikacin. Isolates remained susceptible to nitrofurantoin (the percentage of sensitivity of the strains was 65%). However, isolates were frequently resistant to gentamicin, ciprofloxacin and trimethoprim/sulfamethoxazole with a high MIC90 value (Table 4).

**Table 4:** Antimicrobial susceptibility of ESBL-producing strains

	Minimal inhibitory concentration (μg/ml)*							
**Name of antibiotic**	***Citrobacter freundii *(n = 5)**		***Enterobacter cloacae *(n = 9)**		***Escherichia coli *(n = 41)**		***Klebsiella pneumonia*e (n = 3)**	

	**%R**	**MIC90**	**%R**	**MIC90**	**%R**	**MIC90**	**%R**	**MIC90**

Temocillin	0	2	0	2	0	16	0	8

Meropenem	0	.125	0	.125	0	.125	0	.125

Amikacin	0	16	0	4	0	4	0	1

Gentamicin	100	32	100	32	61	32	100	32

Ciprofloxacin	100	8	75	8	75.6	8	67	8

Nitrofurantoin	0	64	33.3	64	34.1	128	67	256

Trimethoprim/Sulfamethoxazole	100	256	100	256	97.5	256	100	256

* R: resistant isolates

### ß-lactamase characterization

All isolates defined as "presumptive ESBL producers" were confirmed as ESBL producers by PCR-sequencing of ESBL genes. Most of the strains (97%) produced a CTX-M group 1 enzymes [CTX-M-15 (98%) or CTX-M-1 (2%)]. The CTX-M-15 gene was detected alone (20%) or in association with other ß-lactamase genes: OXA-1 and TEM-1 (45%), TEM-1 and SHV-1 (4%), TEM-1 alone (18%), OXA-1 alone (13%); while, CTX-M-1 gene was associated with TEM-1 ß-lactamase gene.

The ESBL SHV-12 (3%) was found in two strains (1 *Citrobacter freundii *and 1 *Escherichia coli*) (Table 5).

**Table 5:** Characterization of ESBL producers among 58 strains isolated from outpatients and student volunteers in faecal flora

	No. of ESBL producers (%)	Positive genes detected by multiplex and simplex PCR assays (no. of isolates)	ESBL (no. of isolates)	Other ß-lactamase genes
**Outpatients**				

*Citrobacter freundii*	5 (10.4)	TEM, OXA-1-like, CTX-M (3)	CTX-M-15 (3)	TEM-1, OXA-1

		TEM, CTX-M (1)	CTX-M-15 (1)	TEM-1

		TEM, SHV(1)	SHV-12 (1)	TEM-1

*Enterobacter cloacae*	9 (18.8)	TEM, OXA-1-like, CTX-M (9)	CTX-M-15 (9)	TEM-1, OXA-1

*Escherichia coli*	32 (66.7)	CTX-M (10)	CTX-M-15 (10)	None

		TEM, CTX-M (8)	CTX-M-15 (7)	TEM-1

			CTX-M-1 (1)	TEM-1

		OXA-1-like, CTX-M (7)	CTX-M-15 (7)	OXA-1

		TEM, OXA-1-like, CTX-M (6)	CTX-M-15 (6)	TEM-1, OXA-1

		SHV (1)	SHV-12 (1)	None

*Klebsiella pneumoniae*	2 (4.2)	CTX-M (1)	CTX-M-15 (1)	None

		TEM, SHV, CTX-M (1)	CTX-M-15 (1)	TEM-1, SHV-1

**Student volunteers**				

*Escherichia coli*	9 (90)	TEM, OXA-1-like, CTX-M (7)	CTX-M-15 (7)	TEM-1, OXA-1

		TEM, CTX-M (2)	CTX-M-15 (2)	TEM-1

*Klebsiella pneumoniae*	1 (10)	TEM, SHV, CTX-M (1)	CTX-M-15 (1)	TEM-1, SHV-1

## Discussion

ESBL-producing *Enterobacteriaceae *have emerged worldwide and have been reported recently in outpatients in many countries. However, in African countries, data on extended-spectrum ß-lactamase (ESBL)-producing *Enterobacteriaceae *are scarce. In Cameroon, since the previous reports of SHV-12 and CTX-M-15 in 2005 [13,16], there were no other reports on the subject in the country and there is no available information regarding the resistance mechanisms of extended-spectrum ß-lactamase (ESBL)-producing *Enterobacteriaceae *in the community. This study determines the proportion of ESBL-producing *Enterobacteriaceae *in the community and analyses some risk factors associated with ESBL carriage. In this study, the proportion of faecal carriage of ESBL-producing *Enterobacteriaceae *among outpatients was 23.1%; this is higher than that observed among student volunteers (6.7%), *p *< 0.000. This value is even higher than those reported in Spain (5.5% in outpatients and 3.7% in healthy volunteers) [5], in China (6.5% in outpatients) [6] and in Saudi Arabia (13.7% in outpatients) [8]. Many factors have been found to contribute to such high rates of resistance in developing countries. These include: poor drug quality or inadequate posology, the long-term treatments, misuse of antibiotics by health professionals, unskilled practitioners, auto medication (antibiotics can be purchased without prescription), unhygienic conditions accounting for the spread of resistant bacteria and inadequate surveillance programs [4,23].

Our study also showed high resistance rates to gentamicin, ciprofloxacin and trimethoprim/sulfamethoxazole but none of the isolates was resistant to temocillin, amikacin or meropenem. Very similar results have been previously found in Cameroon from outpatients and inpatients [24] and in other countries: Morocco [25], Benin [14], Tanzania [15], Ethiopia [26], England [27] and Canada [28].

In addition, the majority of outpatients in this study had been treated with amoxicillin and ciprofloxacin within 3 months prior to inclusion in the study (Table 1). Drugs such as amoxicillin, ciprofloxacin and trimethoprim/sulfamethoxazole represent the first-line antibiotics commonly used by patients without prescription. The presence of ESBL-producing bacteria complicates the selection of antibiotics used for empirical therapy. Other drugs such as temocillin, amikacin or meropenem, which should represent the first-line treatment for infections with ESBL-producing *Enterobacteriaceae*, are very expensive and difficult for the population to obtain in Cameroon. As the available treatment options are limited, antibiotic control policies, together with the implementation of infection control measures, remain therefore of high importance.

In our study, the previous use of fluoroquinolone (ciprofloxacin) appeared to have been a risk factor for ESBL carriage (*p *< 0.05). Fluoroquinolones are potent antimicrobials; they have been in clinical use for the last two decades and have been the most commonly prescribed antibiotic for community-acquired UTIs [29]. An association between the increase in quinolone prescriptions and an increase in bacterial resistance has been reported from several different countries [29,30].

Although, in our study, there were no much records of recent hospitalization or antibiotic consumption among the student volunteers, we found a significant proportion of ESBL-producing bacteria in their faecal flora. For these students, many are likely to have been exposed to multiple courses of antibiotics due to the unrestricted or over-the-counter availability of antibiotics in developing countries [8]. In addition, the increasing prevalence of ESBL producers in the community increases the risk that other individuals will become carriers as a consequence of human-to-human transmission of resistant bacteria or through the environment as well [31].

In the present study, *Escherichia coli *was the most frequent species isolated from both outpatients and student volunteers representing 66.7% and 90% of all isolates, respectively. CTX-M-type enzymes represented 97% of ESBLs detected. CTX-M-15 was predominant and was found to be the most frequent *bla*_ESBL _present in *Escherichia coli, Enterobacter cloacae, Citrobacter freundii *and *Klebsiella pneumoniae*. These findings support the evidence of the dissemination of *bla*_CTX-M-15 _in community setting. Widespread dissemination of CTX-M-15 has been described previously in Cameroon among hospitalized patients [16] and in other places: Portugal [32], Spain [33], England [9], the United States [10], Latin America [11] and China [12]. The strains in our study that produced CTX-M-15 genes were also co-produced TEM-1 and OXA-1 ß-lactamase genes. These results are consistent with previous studies from Canada [28] and France [34]. We also identified type SHV-12 in *Escherichia coli *and *Citrobacter freundii*. This enzyme has risen to prominence in recent years and has been detected in different species of *Enterobacteriaceae *in many parts of the world [5].

Our study has some limitations. Firstly, we did not screen the ESBL positive isolates for the presence of the human pandemic O25:H4-ST131 clonal group. These investigations have now been planned for a future study. Secondly, it would also be interesting to take the study further by investigating the molecular characteristics of the different bacteria studied in order to better understand the spread of resistant bacteria.

Finally, due to some missing data from the questionnaires (some patients subjects did not recall the requested information), not all patients were included in the respective statistical analyses of the different parameters studied.

## Conclusions

The use of drugs such as amoxicillin, ciprofloxacin and trimethoprim/sulfamethoxazole does not seem appropriate for empirical treatment because of emerging resistance. Our results highlight the presence and spread of *bla *_CTX-M _in the community and confirm that CTX-M-producing *Escherichia coli *is the most frequent bacterial species isolated in the community. These findings underline the need for the rational use of antibiotics as well as for infection control measures. The implementation in Cameroon or in other African countries of methods of screening ESBL-producing organisms in routine laboratories is of great importance in order for us to offer patients appropriate treatment and for infection control efforts to succeed. We believe that our results could provide useful information for clinical purposes and in the fight against bacterial resistance to antibiotics in Cameroon.

## Competing interests

The authors declare that they have no competing interests.

## Authors' contributions

CML was the principal investigator and participated in the planning and execution of the study. CM and RB participated in the planning of the molecular biology work. JGP participated in data analysis and in the reading of the manuscript. MCO was the co-director of the thesis and participated in the supervision of activities in Cameroon and in the reading of the manuscript. PM provided advice and participated in the writing of the manuscript. PD was the director of thesis and participated in both planning and writing. All authors have read and approved the final manuscript.

## Pre-publication history

The pre-publication history for this paper can be accessed here:

http://www.biomedcentral.com/1471-2334/12/53/prepub
